# Dissection of the style’s response to pollination using transcriptome profiling in self-compatible (*Solanum pimpinellifolium*) and self-incompatible (*Solanum chilense*) tomato species

**DOI:** 10.1186/s12870-015-0492-7

**Published:** 2015-05-15

**Authors:** Panfeng Zhao, Lida Zhang, Lingxia Zhao

**Affiliations:** Joint Tomato Research Institute, School of Agriculture and Biology, Shanghai Jiao Tong University, Shanghai, 200240 China; Plant Biotechnology Research Center, School of Agriculture and Biology, Shanghai Jiao Tong University, Shanghai, 200240 China

**Keywords:** Tomato, Self-incompatibility, Self-compatibility, Style, Transcriptome

## Abstract

**Background:**

Tomato (*Solanum lycopersicum*) self-compatibility (SC) is defined as self-pollen tubes that can penetrate their own stigma, elongate in the style and fertilize their own ovules. Self-incompatibility (SI) is defined as self-pollen tubes that are prevented from developing in the style. To determine the influence of gene expression on style self-pollination, a transcriptome-wide comparative analysis of SC and SI tomato unpollinated/pollinated styles was performed using RNA-sequencing (RNA-seq) data.

**Results:**

Transcriptome profiles of 24-h unpollination (UP) and self-pollination (P) styles from SC and SI tomato species were generated using high-throughput next generation sequencing. From the comparison of SC self-pollinated and unpollinated styles, 1341 differentially expressed genes (DEGs) were identified, of which 753 were downregulated and 588 were upregulated. From the comparison of SI self-pollinated and unpollinated styles, 804 DEGs were identified, of which 215 were downregulated and 589 were upregulated. Nine gene ontology (GO) terms were enriched significantly in SC and 78 GO terms were enriched significantly in SI. A total of 105 enriched Kyoto Encyclopedia of Genes and Genomes (KEGG) pathways were identified in SC and 80 enriched KEGG pathways were identified in SI, among which “Cysteine and methionine metabolism pathway” and “Plant hormone signal transduction pathway” were significantly enriched in SI.

**Conclusions:**

This study is the first global transcriptome-wide comparative analysis of SC and SI tomato unpollinated/pollinated styles. Advanced bioinformatic analysis of DEGs uncovered the pathways of “Cysteine and methionine metabolism” and “Plant hormone signal transduction”, which are likely to play important roles in the control of pollen tubes growth in SI species.

**Electronic supplementary material:**

The online version of this article (doi:10.1186/s12870-015-0492-7) contains supplementary material, which is available to authorized users.

## Background

In flowering plants, the male organ of the flower is the stamen and the female organ of the flower is pistil. The stamen comprises an anther generating pollen grains and a filament supporting the anther. The pistil comprises the stigma, the style and the ovary. Pollination is a process of pollen-pistil interaction during which pollen adheres, hydrates, and germinates on the stigma, the pollen tube elongates on an active extracellular matrix in the style and finally transports male gametes (sperm cells) to the ovary, releasing it into ovules to complete fertilization [1]. Mate selection is crucial to successful reproduction and species survival [2]. Self-compatibility (SC) and self-incompatibility (SI) are the two predominant forms of mate selection. SC is defined as self-pollen that can penetrate its own pistil and fertilize its own ovules [1]; SI is where self-pollen is prevented from developing on the pistil [3].

Tomatoes (*Solanum lycopersicum*) are one of the most important vegetable crops in the world, and possess genetic diversities in fruit color, size, and mating system. In particular, the mating systems play key roles to control the reproductive habits between intra-/interspecies in tomatoes. Generally, color-fruited species such as *Solanum lycopersicum*, *S. pimpinellifolium* and *S. neorickii* are SC species, while some green-fruit species, such as *S. habrochaites* and *S. chilense*, are SI species [4]. However, the growth of pollen tubes within styles differs between SI and SC species. Pollen growth is arrested at the middle style in SI species, but not in SC. Some models were proposed for growth behavior of pollen tubes within styles that are related to pollen factors such as F-box protein and pistil factor of RNase [5,6]; however, the mechanism controlling the growth of pollen tubes remains unclear in tomatoes.

The transcriptome is the sum of all the RNA transcription for specific cells in a certain functional condition, including mRNAs, non-coding RNAs (ncRNA) and small RNAs [7,8]. RNA-Seq is a deep-sequencing technology [7,9] that has many advantages compared with Serial Analysis of Gene Expression (SAGE) [10], Expressed Sequence Tag (EST) [11], cDNA-amplified fragment length polymorphism (AFLP) [12], DNA microarrays [13] and massively parallel signature sequencing (MPSS) [14]. RNA-seq has already been widely used for transcriptome research in *Miscanthus sinensis* [15], tomato [16], *Wolfiporia cocos* [17], *Hevea brasiliensis* [18], *Populus tomentosa* [19], *Lolium rigidum* [20] and wheat [21]. It has also been applied to study pollination in maize [22,23], and to study SC/SI in *Citrus clementina* [24], lemon [25] and *Leymus chinensis* [26]. To understand what occurs after pollination in the styles of tomatoes of different mating types at the transcriptome level, we compared the transcription profiles differences between tomato SI and SC species. The results provide valuable information for understanding the growth behavior of pollen tubes within styles.

At present, research into tomato SC and SI has mainly concentrated on the S-RNase aspect, with no comprehensive transcriptome-level studies. Thus, to the best of our knowledge, this is the first study to perform comparative transcriptome analyses of SC and SI tomato unpollinated/pollinated styles using RNA-seq. The results of RNA-seq were analyzed by mapping, differential gene expression analysis, GO and pathway analysis. The results revealed comprehensive information concerning SI and SC, and provided clues to the molecular mechanisms of SI and SC.

## Results

### Summary of RNA-seq datasets

SC unpollination/self-pollination (SCUP/SCP) and SI unpollination/self-pollination (SIUP/SIP) styles **(**total of 12 samples**)** were performed RNA-seq. The raw sequence data yielded approximately 3.0 gigabases (GB) per sample and more than 96% of the raw read pairs obtained had a quality score of ≥ Q20. Total raw read pairs among the 12 samples ranged from 15 to 18 million. By later removing reads containing adapters, reads containing poly-N and low-quality reads from the raw data, high-quality read pairs were obtained. The number of high-quality read pairs among the 12 samples ranged from 14 to 17 million (about 98% of the raw read pairs). Approximately 90% of the high-quality read pairs from the SC samples and 70% of the SI samples could be mapped to the tomato reference genome sequence. In addition, unmapped read pairs ranged from 1 to 5 million and multiple mapped read pairs ranged from about 0.30% to 0.50% of mapped read pairs among the 12 samples (Table 1).

**Table 1:** **Statistics of raw and mapped read pairs from RNA**-**seq analysis of SC unpollination/self-pollination (SCUP/SCP) and SI unpollination/self-pollination (SIUP/SIP) styles**

**Sample ID**	**Raw read pairs**	**High-quality read pairs**	**High-quality Percent**	**Mapped read pairs**	**Mapped Percent**	**Unmapped read pairs**	**Multi-mapped read pairs**	**Multi-mapped Percent**
SCP1	17000933	15215933	89.50%	13817410	90.80%	1398523	65920	0.50%
SCP2	16374027	14680679	89.66%	13485391	91.90%	1195288	59339	0.50%
SCP3	17667649	15893802	89.96%	14489321	91.20%	1404481	67431	0.50%
SCUP1	18248702	16320337	89.43%	14747316	90.40%	1573021	48233	0.30%
SCUP2	17346914	15557760	89.69%	14145517	90.90%	1412243	59543	0.40%
SCUP3	18986356	17021427	89.65%	15362024	90.30%	1659403	56730	0.40%
SIP1	15510971	13879490	89.48%	9431478	68.00%	4448012	32428	0.30%
SIP2	16845976	15163409	90.01%	10608995	70.00%	4554414	37544	0.40%
SIP3	16920459	15154474	89.56%	10396040	68.60%	4758434	43009	0.40%
SIUP1	17664280	15847493	89.71%	10885898	68.70%	4961595	29071	0.30%
SIUP2	17752773	15880716	89.45%	11004025	69.30%	4876691	31678	0.30%
SIUP3	18253204	16435260	90.04%	11232677	68.30%	5202583	31212	0.30%

### Differential gene expression profiles of unpollinated (UP) and self-pollinated (P) styles in SC and SI, and hierarchical cluster analysis

**Figure 1:**
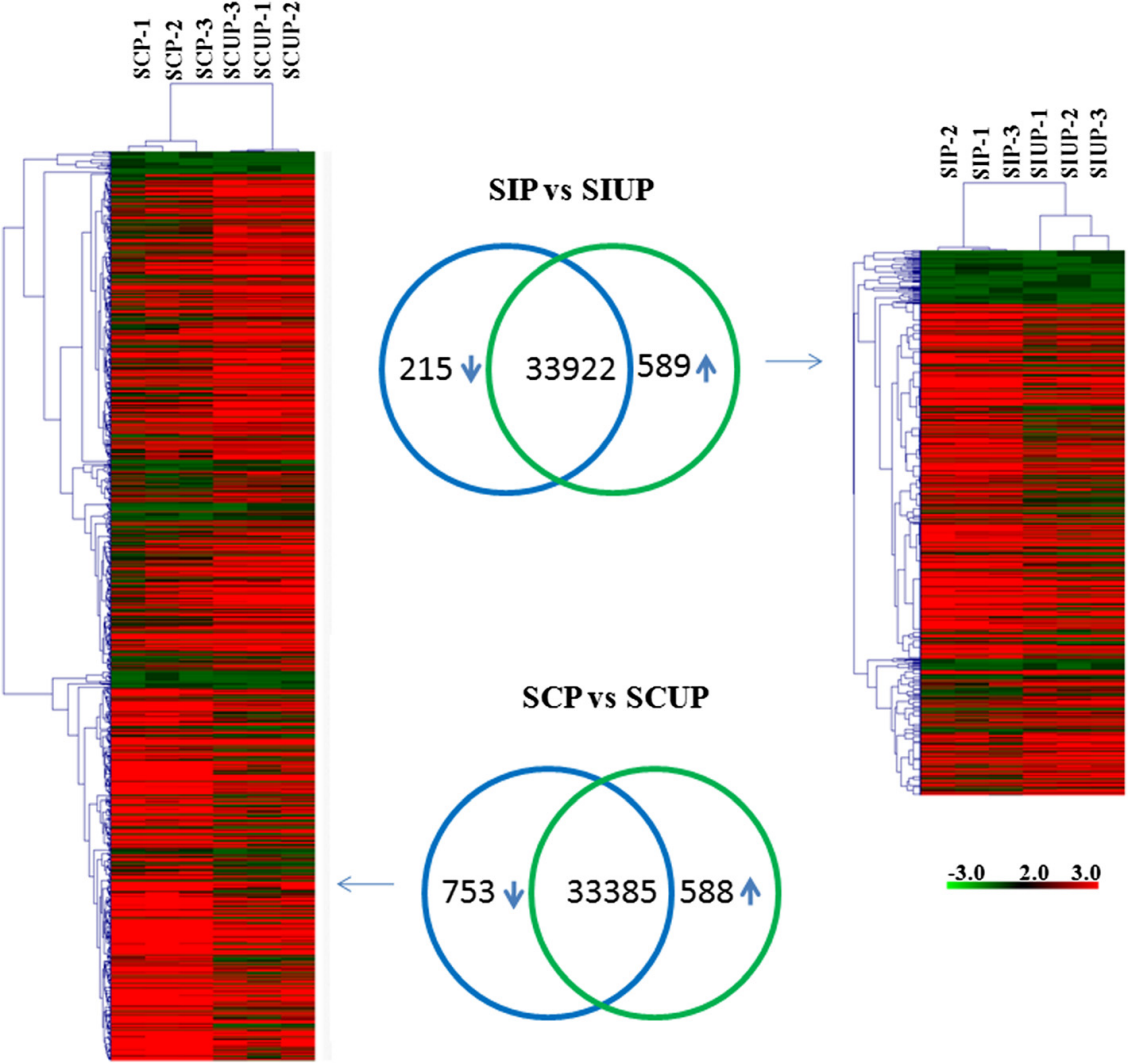
Clustering of differentially expressed genes in unpollination (UP) and pollination (P) styles in SC and SI.

To quantify the expression levels of the transcripts, HT-seq was used to count the read numbers mapped to each gene, based on the 34,726 genes of the tomato reference genome. These data were then normalized to reads per kilobase of exon region in a given gene per million mapped reads (RPKM) values, which were calculated based on the length of the gene and read count mapped to this gene. The RPKM values for each gene are listed in Additional file 1. To determine differential expression genes (DEGs) of UP and P styles in SC and SI, we screened for DEGs between UP and P styles in SC, and between UP and P styles in SI using the following criteria: Log_2_ fold-change (FC) > 1 or Log_2_FC < −1 and P-value < 0.05. We identified 1341 DEGs between UP and P styles in SC, and 804 DEGs between UP and P styles in SI (Additional file 2). Of these DEGs, 753 genes were downregulated and 588 genes were upregulated after self-pollination in SC; 215 genes were downregulated and 589 genes were upregulated after self-pollination in SI (Figure 1). We used hierarchical cluster analysis to compare the DEGs between UP and P styles in SC, between UP and P styles in SI, and the similarity of the expression patterns of the three biological replicates (Figure 1).

### GO annotation of all DEGs in SCP *vs*. SCUP and SIP *vs*. SIUP

**Figure 2:**
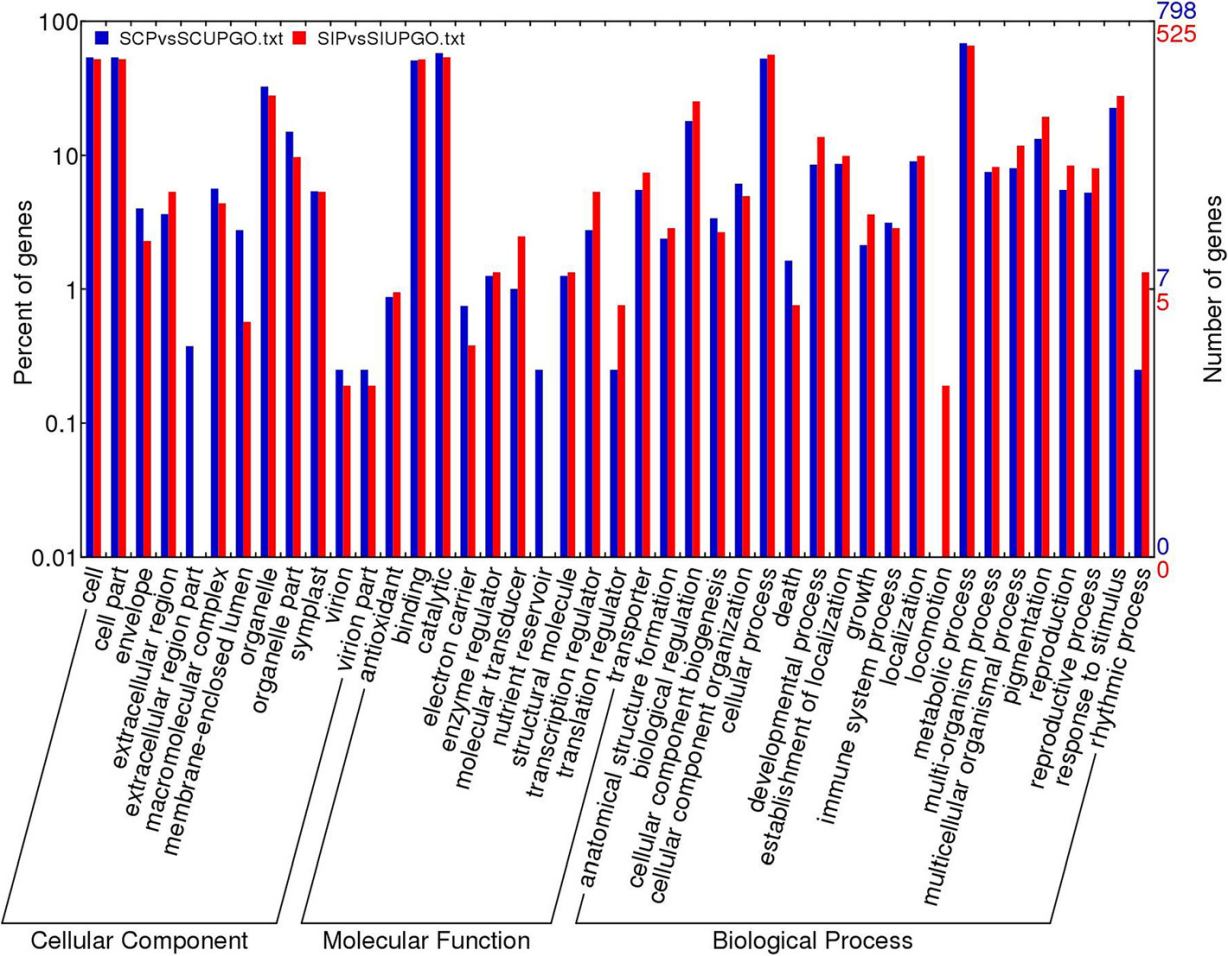
GO assignment and comparison of all DEGs in SCP *vs*. SCUP and SIP *vs*. SIUP. All DEGs in SCP *vs*. SCUP and SIP *vs*. SIUP were annotated in three main categories: biological processes, cellular components and molecular functions. The left and right hand y-axes indicate the percentage and the number of annotated genes in each category, respectively.

To identify the functions of thee DEGs, we performed gene ontology (GO) analysis. A total of 798 DEGs of SC comparing UP and P styles were assigned GO annotations and 525 DEGs of SI comparing UP and P styles were assigned GO annotations. GO has three ontologies: molecular function, cellular component and biological process. In many cases, one gene was annotated with multiple GO terms. The GO terms of 798 DEGs of SCP *vs*. SCUP styles were categorized into 42 main functional groups belonging to the three categories and the GO terms of 525 DEGs of SIP *vs*. SIUP styles were categorized into 41 main functional groups belonging to the three categories (Figure 2).

### Comparative analysis of GO terms assigned to SCP *vs*. SCUP DEGs and those assigned to SIP *vs*. SIUP DEGs

To better understand the distribution of gene functions at the macro level, the GO function classification of the DEGs in SCP *vs.* SCUP styles and SIP *vs*. SIUP styles were analyzed using the WEGO online tool. The comparative analysis showed that DEGs in SCP *vs.* SCUP styles and SIP *vs.* SIUP styles shared broad similarities in the proportion of genes in the three main categories, but differences were detected in many subcategories (Figure 2). Most GO terms of DEGs in SCP *vs.* SCUP styles and SIP *vs.* SIUP styles were categorized into the same biological processes, cellular components and molecular functions. Most GO subcategories terms were detected in both of SCP *vs.* SCUP styles and SIP *vs.* SIUP styles; however, GO subcategory terms, including membrane-enclosed lumen, organelle part, molecular transducer, transcription regulator, biological regulation, developmental process, multicellular organismal process, pigmentation, reproduction, reproductive process and response to stimulus showed significant (P-value < 0.05) differences in counts between SCP *vs.* SCUP styles and SIP *vs.* SIUP styles. These results suggested that despite certain mechanisms of SC and SI appear to be conserved, the regulation mechanisms appear to be different between these two reproductive systems.

### GO enrichment analysis of all DEGs in SCP *vs*. SCUP and SIP *vs*. SIUP

**Figure 3:**
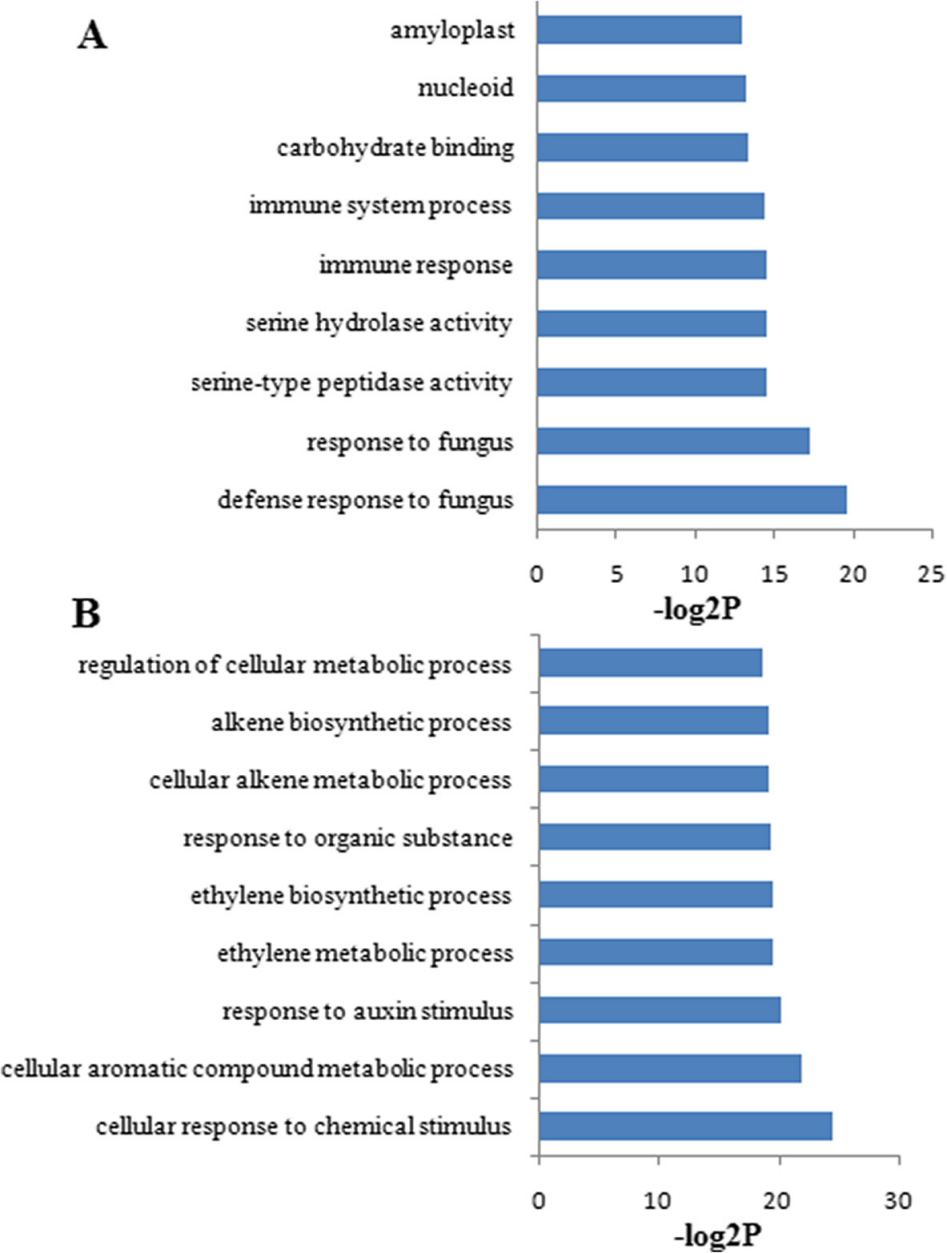
Significant gene ontology analysis of DEGs in SCP *vs*. SCUP and SIP *vs*. SIUP. **A**. Significant GO terms of SCP *vs*. SCUP; **B**. Significant GO terms of SIP *vs*. SIUP (The first nine significant GO terms). P-value < 0.05 and FDR < 0.05 for all significant GO terms.

Significantly enriched GO terms were identified using singular enrichment analysis (SEA). The results showed that nine GO terms were significant in DEGs of SCP *vs.* SCUP based on a P-value < 0.05 and the false discovery rate (FDR) < 0.05 cutoffs (Figure 3A), which comprised two, three and four terms for the cellular components, molecular functions, biological processes categories, respectively. Seventy-eight GO terms were significant in DEGs of SIP *vs.* SIUP based on a P-value < 0.05 and the FDR < 0.05 cutoffs (Figure 3B, only 9), which comprised eight and 70 terms for the molecular functions and biological processes categories, respectively. The detailed results of the SCP *vs.* SCUP and SIP *vs.* SIUP Go enrichment analysis are presented in Additional file 3.

### KEGG pathway mapping of all DEGs in SCP *vs*. SCUP and SIP *vs*. SIUP

**Figure 4:**
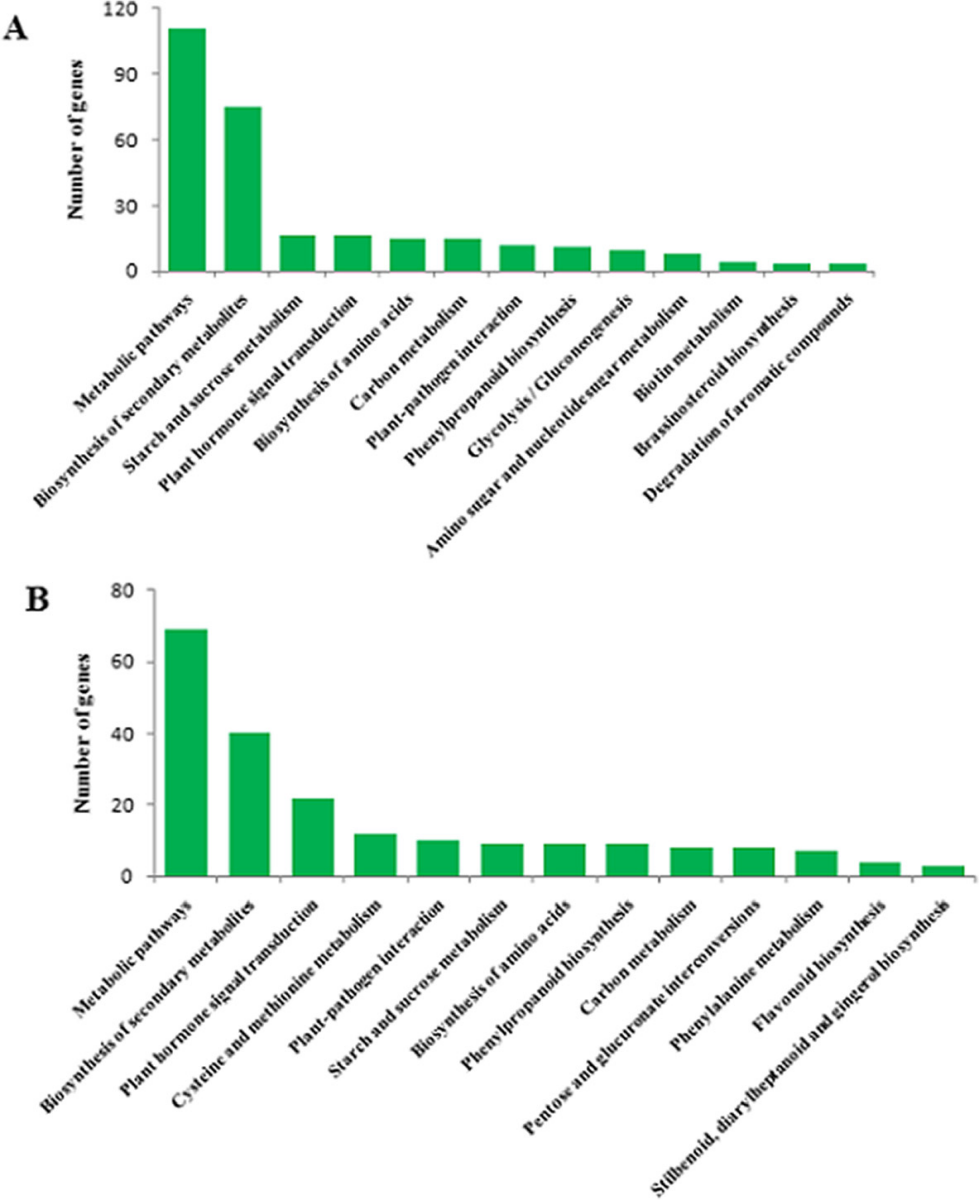
Pathway enrichment analysis of DEGs in SCP *vs*. SCUP and SIP *vs*. SIUP based on KEGG. **A**. Enriched pathways in SCP *vs*. SCUP; **B**. Enriched pathways in SIP *vs*. SIUP.

**Figure 5:**
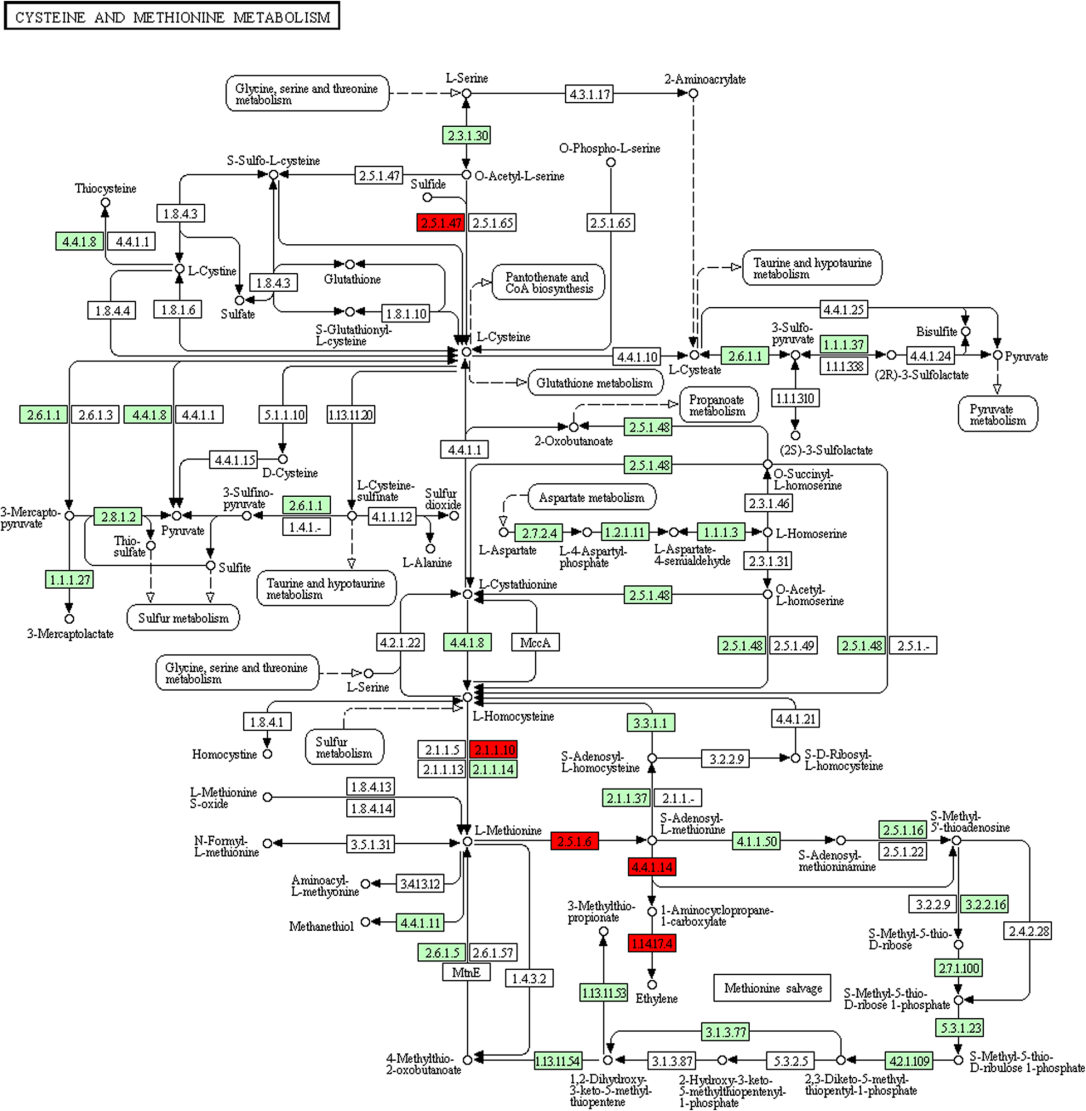
Expression features of cysteine and methionine metabolism pathway genes. Red boxes represent tomato genes that were identified as differentially expressed in SI compared with pollinated and unpollinated styles. Light green boxes represent genes that have been previously identified in tomatoes. White boxes represent genes that belong to the KEGG pathway, but have not been identified in tomatoes until now.

**Figure 6:**
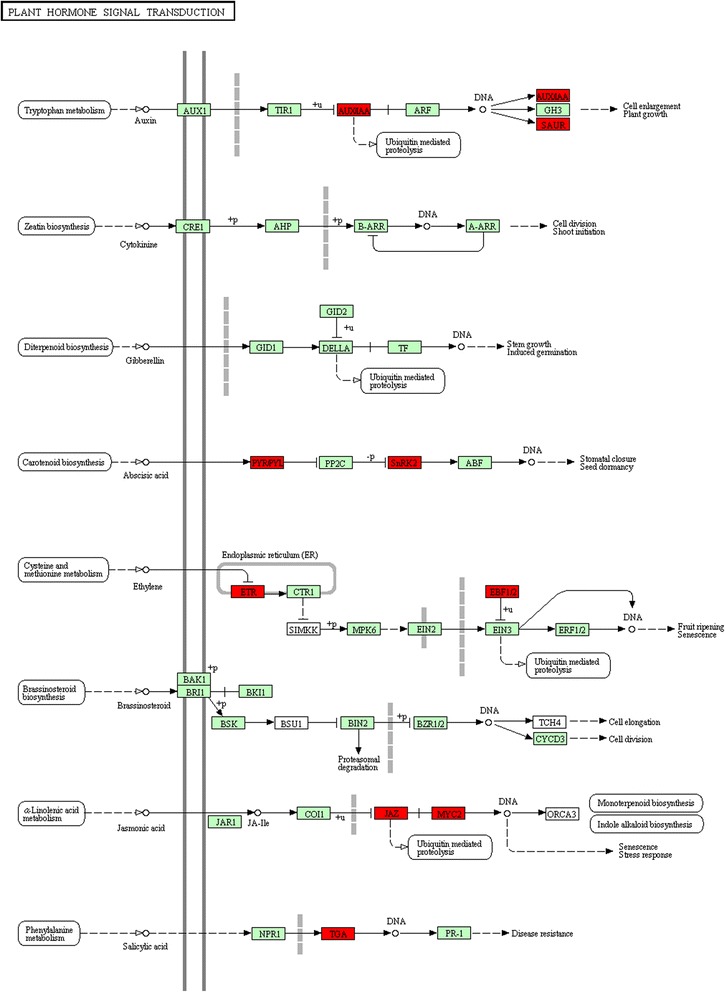
Expression features of plant hormone signal transduction pathway genes. Red boxes represent tomato genes that were identified as differentially expressed in SI compared with pollinated and unpollinated styles. Light green boxes represent genes that have been previously identified in tomatoes. White boxes represent genes that belong to the KEGG pathway, but have not been identified in tomatoes until now.

To further investigate the influence of the DEGs on pathways, statistical pathway enrichment analysis of DEGs in SCP *vs.* SCUP and SIP *vs.* SIUP were performed based on KEGG database, using Fisher’s exact test. The DEGs of SCP *vs.* SCUP were enriched in 105 KEGG metabolic pathways and the DEGs of SIP *vs.* SIUP were enriched in 80 KEGG metabolic pathways (Additional file 4). The top ten KEGG metabolic pathways and three P-value < 0.05 metabolic pathways of the DEGs in SCP *vs.* SCUP are shown in Figure 4A. Among these 105 pathways of SCP *vs.* SCUP, those containing the greatest numbers of DEGs transcripts were “Metabolic pathways” (containing 111 DEGs) and “Biosynthesis of secondary metabolites” (containing 75 DEGs). Other GO terms associated with higher numbers of DEGs were “Starch and sucrose metabolism” (16 DEGs), “Plant hormone signal transduction” (16 DEGs), “Biosynthesis of amino acids” (15 DEGs), “Carbon metabolism” (15 DEGs), “Plant-pathogen interaction” (12 DEGs), “Phenylpropanoid biosynthesis” (11 DEGs), “Glycolysis/Gluconeogenesis” (nine DEGs), and “Amino sugar and nucleotide sugar metabolism” (eight DEGs); The pathways of “Biosynthesis of secondary metabolites”, “Biotin metabolism”, “Brassinosteroid biosynthesis” and “Degradation of aromatic compounds” had P-values < 0.05 (Figure 4A). For SIP *vs.* SIUP, of 13 KEGG metabolic pathways were identified. The top 11 KEGG metabolic pathways and two P-value < 0.05 metabolic pathways of DEGs in SIP *vs.* SIUP are shown in Figure 4B. Among the 80 pathways of SIP *vs.* SIUP, those containing the greatest numbers of DEGs were “Metabolic pathways” (69 DEGs), “Biosynthesis of secondary metabolites” (40 DEGs), “Plant hormone signal transduction” (22 DEGs), “Plant-pathogen interaction” (10 DEGs), “Starch and sucrose metabolism” (9 DEGs), “Biosynthesis of amino acids” (nine DEGs), “Phenylpropanoid biosynthesis” (nine DEGs), “Carbon metabolism” (eight DEGs), “Pentose and glucuronate interconversions” (eight DEGs), “Phenylalanine metabolism” (seven DEGs). The pathways of “Cysteine and methionine metabolism”, “Plant hormone signal transduction”, “Pentose and glucuronate interconversions”, “Flavonoid biosynthesis” and “Stilbenoid, diarylheptanoid and gingerol biosynthesis” all had P-values < 0.05 (Figure 4B). In addition, the pathways of “Cysteine and methionine metabolism” and “Plant hormone signal transduction” were significant pathways in DEGs of SIP *vs.* SIUP, based on a P-value < 0.05 and the FDR < 0.05 cutoffs (Figure 4B). The detailed results of the SIP *vs.* SIUP significant pathways enrichment analysis are presented in Figures 5 and 6.

“Cysteine and methionine metabolism” is the ethylene biosynthesis pathway, which was significantly enriched in the SIP *vs.* SIUP analysis. DEGs were enriched in the step of O-Acetyl-L-serine conversion to L-Cysteine, L-Homocysteine conversion to L-Methionine, L-Methionine conversion to S-adenosyl-L-methionine (AdoMet), AdoMet conversion to 1-aminocyclopropane-1-carboxylate (ACC) and ACC production ethylene (Figure 5). L-Methionine conversion to AdoMet was the first step of ethylene biosynthesis, AdoMet conversion to ACC was the rate-limiting step in ethylene biosynthesis and ACC production ethylene was the last steps for ethylene biosynthesis. Plant hormone signal transduction is very important to hormone-instigated biochemical changes during plant growth, development, and environmental information processing pathways, which were also significantly enriched in the SIP *vs.* SIUP comparison. DEGs were also enriched in Auxin signal transduction, Abscisic acid (ABA) signal transduction, Ethylene signal transduction, Jasmonic acid (JA) signal transduction and Salicylic acid (SA) signal transduction (Figure 6).

**Figure 7:**
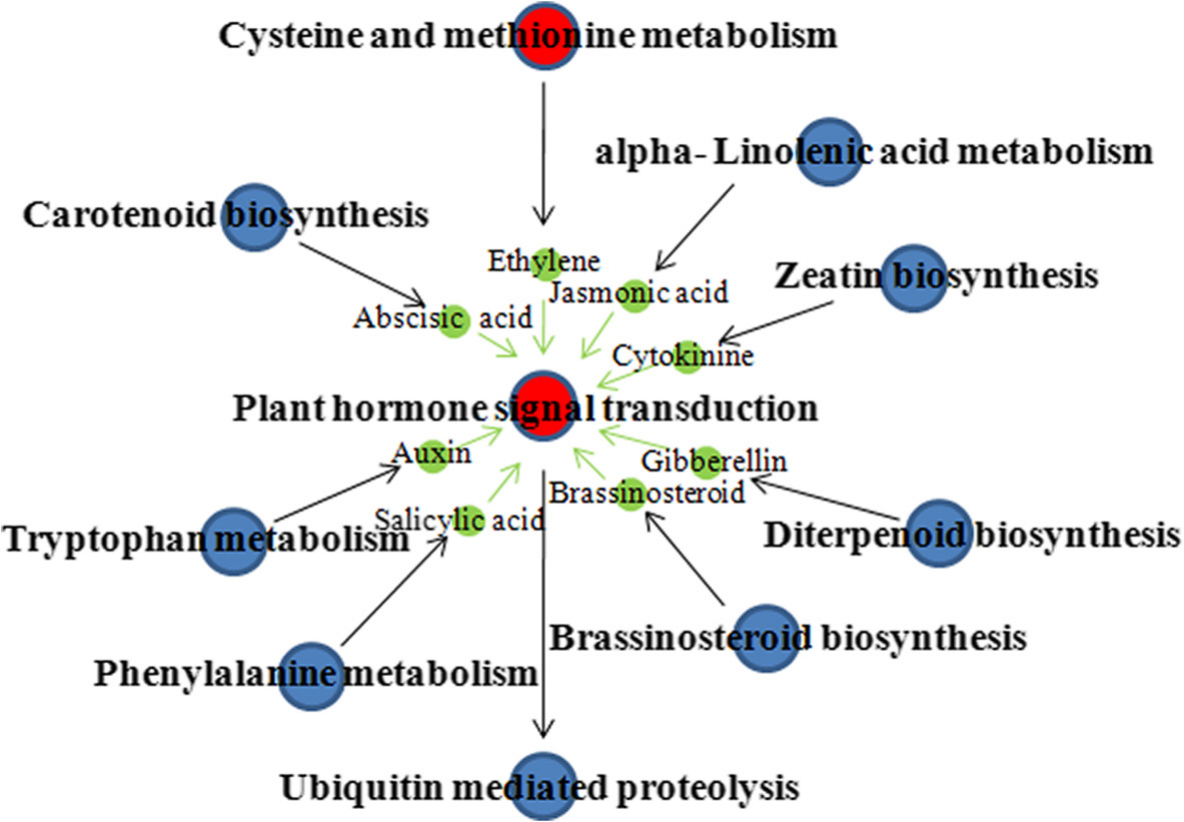
Significant pathways enrichment analysis and interaction network of SIP *vs*. SIUP based on KEGG. Red circles represent significantly enriched pathways.

Significant pathways enrichment analysis showed that cysteine and methionine metabolism and plant hormone signal transduction were the most important pathways in SIP *vs.* SIUP comparison, and plant hormone signal transduction was the key biological event. All the plant hormone signaling pathways pointed to it and the significant pathway of “Cysteine and methionine metabolism” also (Figure 7). This evidence indicated that plant hormone signal transduction plays important roles in tomato SI.

## Discussion

RNA-seq is a powerful tool that can provide a global overview of gene expression at the transcriptome level. With reductions in sequencing costs and the advance of technologies, RNA-seq will become more accessible to researchers to identify and track the expression changes of all genes [7]. The present study identified 1341 significant (P-value < 0.05) DEGs after comparing UP and P styles in SC and 804 significant (P-value < 0.05) DEGs in the comparison of UP and P styles in SI, using RNA-seq analysis. The total number of gene changes demonstrated that SC self-pollination and SI self-pollination are complex processes. This finding is consistent with other plant pollination studies. For example, 1025 differentially expressed genes were potentially involved in the pollination response and SI mechanisms in sheepgrass [26]. In a comparison of pollinated and unpollinated stigmas with styles, 4785 DEGs were identified in SI lemon [25]. These data demonstrate the complex nature of the transcriptome changes in SC self-pollination and SI self-pollination.

Pollination shares striking similarities with fungal infection in terms of biological responses and processes that result in cell death [27,28]. Our transcriptome GO enrichment analysis identified several significant GO terms involved in pathogen invasion responses, such as defense response to fungus, response to fungus, immune response, and immune system process in the SCP *vs.* SCUP comparison. This result is consistent with other plant pollination studies, such as in Arabidopsis [29,30] and rice [31]. However, GO terms involved in stimuli and hormones were the most important of the 78 significant GO terms in the SIP *vs.* SIUP comparison.

Pollination leads to senescence of petunia corollas by inducing many hormonal, physiological, and molecular changes [32]. Ethylene is a gaseous plant hormone with a wide range of effects on plant growth and development [33]. Ethylene is synthesized from L-Methionine via the intermediates AdoMet and ACC (Figure 5) [34-36]. AdoMet is made from L-Methionine by the enzyme S-adenosylmethionine synthase (SAM), representing the first step of ethylene biosynthesis (Figure 5). 1-aminocyclopropane-1-carboxylate synthase (ACS) gene family members and 1-aminocyclopropane-1-carboxylate oxidase (ACO) gene family members are two important enzymes for ethylene biosynthesis. ACS catalyzes the conversion of AdoMet to ACC, which is the rate-limiting step in ethylene biosynthesis. ACO then catalyzes the conversion of ACC to ethylene (Figure 5) [37]. After SI self-pollination, one SAM gene (S-adenosylmethionine synthase 2-like) (Solyc10g083970), five ACS gene family members (Solyc00g095760, Solyc08g081550, Solyc08g008100, Solyc08g081540, Solyc00g095860) and four ACO gene family members (Solyc02g036350, Solyc07g026650, Solyc07g049530, Solyc07g049550) were significantly upregulated, which indicated that SI self-pollination is associated with results in significant upregulation of ethylene biosynthesis related genes and ethylene production. It has been reported that ethylene biosynthesis is induced by pollination in petunias [38]. After SC self-pollination, although the pathway of “Cysteine and methionine metabolism” was not a significant enrichment pathway in the SCP *vs.* SCP comparison, two ACS gene family members (Solyc08g081540, Solyc00g095860) and one ACO gene family member (Solyc07g049530) were significantly upregulated, which indicated that SC self-pollination results in some upregulation of ethylene biosynthesis of partly related genes. The above results suggest that SI self-pollination induces more ethylene production than SC self-pollination.

Plant hormone signal transduction is very important to hormone triggered biochemical changes [39]. Plant hormone signal transduction plays an important role in pollination of petunias pollination; for example, RNA-seq revealed that plant hormone signal transduction-related KEGG pathways were enriched in petunia corollas when comparing pollinated and unpollinated samples [32]. After SI self-pollination, plant hormone signal transduction-related KEGG pathways were significantly enriched in the SIP *vs.* SIUP comparison, but not after SC self-pollination (Figure 6). This result indicated that plant hormone signal transduction might play an important role in tomato SI. Plants recognize and transduce the ethylene signal via ethylene receptors (ETR) [40] in the ethylene signal transduction pathway (Figure 6) [41]. We identified two ethylene receptors, LeETR6 (Solyc06g053710) and tETR (Solyc09g089610), which were significantly upregulated in the SIP *vs.* SIUP comparison, both of which mapped to the plant hormone signal transduction KEGG pathway. LeETR2 (Solyc07g056580) was the only ethylene receptor identified from the SCP *vs.* SCUP comparison, and significantly downregulated in P styles compared with UP styles. This protein also mapped to the plant hormone signal transduction KEGG pathway, which was not a significantly enriched pathway in the SCP *vs.* SCUP comparison. The above results indicated that SI self-pollination not only involves the induction of ethylene production, but also enhanced the perception ethylene. Although SC self-pollination may involve some enhancement of ethylene production, the ability to perceive ethylene was weakened by the significant downregulation of LeETR2. Plant responses to ethylene initiates with ethylene binding to ETRs and terminates in a transcription cascade of plant-specific transcription factors families, especially the ethylene-insensitive protein 3 (EIN3/EIL) and ethylene-responsive transcription factor (ERF). EIN3 protein is a key transcription factor for mediating the expression of ethylene-regulated genes and morphological responses. EIN3 interacts physically with the Ein3-binding f-box protein1/2 (EBF1/EBF2) and is ultimately and quickly degraded through a ubiquitin/proteasome pathway mediated by the SCF complex, which comprises a RING-box protein 1 (RBX1), Cullin 1 (Cul1), S-phase kinase-associated protein 1 (Skp1), F-box protein (F-box) [42,43]. We identified one EBF1/2 (Solyc07g008250) from the SC and two EBF1/2 (Solyc07g008250, Solyc12g009560) from the SI, both of which were significantly upregulated in P compared with UP styles. In addition, we also identified one Skp1 (Solyc01g111640) and one Cul1 (Solyc01g067120) from SI, which were significantly upregulated in P compared with UP styles. This result indicated that key transcription factor EIN3 was negatively regulated by targeting EIN3 it for degradation through the ubiquitin/proteasome pathway after SI self-pollination, but not in SC pollination.

A previous study demonstrated that auxin was significantly increased after compatible pollination and ethylene was strongly increased after incompatible pollination [44,46]. The last step of indole-3-acetic acid (IAA) biosynthesis is performed by aldehyde dehydrogenase. We identified one aldehyde dehydrogenase (aldehyde dehydrogenase family 2 member B4, Solyc08g068190) from SC that was significantly upregulated in P compared with UP styles and one aldehyde dehydrogenase (aldehyde dehydrogenase family 3 member H1-like, Solyc06g060250) from SI that was significantly downregulated in P compared with UP styles. This result is consistent with the results of the previous study. Auxin is likely to be directly or indirectly involved in pollen-pistil recognition and pollen tube elongation in Nicotiana [45] and might have an important role in the SI response in plants such as *Theobroma cacao* [46], *Petunia hybrida* [47] and *Olea europaea* [48]. Auxins regulate plant growth and development by a complex signal transduction network [49], which was included in the significantly enriched KEGG pathways of plant hormone signal transduction KEGG in the SIP *vs.* SIUP comparison. Auxin influx carrier (AUX1 LAX family) is a polar auxin transporter in cells that is involved in attaining a hormone maximum (Figure 6) [50]. After SC self-pollination, LAX2 protein (auxin influx carrier, AUX1 LAX family) (Solyc01g111310) was significantly downregulated. Auxins alter three major gene families: auxin/indole-3-acetic acid (Aux/IAA), GH3 and small auxin-up RNA (SAUR) to direct plant growth and development (Figure 6) [49,51]. Aux/IAA gene families: IAA1 (Solyc09g083280), IAA2 (Solyc06g084070), IAA3 (Solyc09g065850), IAA19 (Solyc03g120380), IAA22 (Solyc06g008580), IAA26 (Solyc03g121060), IAA35 (Solyc07Vg008020) and IAA36 (Solyc06g066020) were significantly upregulated in the SIP *vs.* SIUP comparison, and only IAA2 (Solyc06g084070), IAA29 (Solyc08g021820) and IAA 35 (Solyc07g008020) were significantly upregulated in the SCP *vs.* SCUP comparison. For the GH3 gene families, only one probable indole-3-acetic acid-amido synthetase GH3.1-like gene (Solyc02g092820) was significantly upregulated in the SCP *vs.* SCUP comparison. For the SAUR gene families, small auxin-up protein 58 (Solyc06g053260), auxin-induced protein 10A5-like (Solyc03g033590), uncharacterized LOC101249064 (Solyc03g124020) and uncharacterized LOC101254455 (Solyc12g009280) were significantly upregulated, and auxin-induced protein 15A-like (Solyc01g110570) and auxin-induced protein 10A5-like (Solyc01g110560) were significantly downregulated in the SIP *vs.* SIUP comparison. Only auxin-induced protein 15A-like (Solyc09g009980) and indole-3-acetic acid-induced protein ARG7-like (Solyc04g081250) were significantly upregulated in the SCP *vs.* SCUP comparison. These results indicated that although auxin was strongly increased after compatible pollination, because the auxin influx carrier (AUX1 LAX family) (Solyc01g111310) was significantly downregulated, fewer auxin-responsive genes showed altered expressions. During SC pollination, the auxin influx carrier (AUX1 LAX family) was not affected, resulting in many auxin-responsive genes showing altered expression after incompatible pollination. A previous study indicated that auxin influx carriers (AUX1 LAX family) were involved in auxin-ethylene interactions in *Arabidopsis thaliana* [52]; however, whether auxin influx carriers (AUX1 LAX family) are also involved in auxin-ethylene interactions in tomato SI is unknown.

Ethylene and JA, as well as ABA and auxin, have direct or indirect interactions [32], but the roles of JA and ABA in tomato pollination, especially in SI self-pollination, were unknown. ABA is a phytohormone that acts in seed dormancy, plant development and environmental stress. The carotenoid biosynthesis pathway is an ABA biosynthesis pathway (Figure 6) that was enriched in SC and SI. Endogenous ABA levels are regulated by both ABA biosynthesis and ABA catabolism: xanthoxin dehydrogenase is a key enzyme for ABA biosynthesis and ABA 8′-hydroxylase is a key enzyme for ABA catabolism [53,54]. Xanthoxin dehydrogenase (Solyc12g056600) was significantly upregulated in SC not in SI and ABA 8′-hydroxylase 1-like (CYP707A2, Solyc08g005610) was significantly upregulated in SI but not in SC, which indicated that endogenous ABA levels increased in SC and decreased in SI styles during pollination. Pyrabactin resistance/pyrabactin resistance-like (PYR/PYL) family is an ABA receptor that is very important to ABA recognition and signaling [55,56]. We identified two genes of the PYR/PYL family: ABA receptor PYL8-like (Solyc03g007310) from SI and ABA receptor PYL6-like (Solyc06g050500) from SC. PYL8-like was significantly downregulated in SI and PYL6-like was significantly upregulated in SC styles during pollination, which indicated that the ability to perceive ABA was weakened in SI and enhance in SC. A previous study showed that PYR/PYLs are negative regulatory receptors, whereby ABA binds to PYR/PYLs, which in turn binds to type 2C protein phosphatases (PP2Cs) to inhibit PP2Cs. SNF1-related protein kinase subfamily 2 (SnRK2) is located downstream of PP2Cs and is negatively regulated by PP2Cs (Figure 6). SnRK2 (serine/threonine-protein kinase SAPK3-like, Solyc08g077780) was upregulated in SI (in which PP2Cs are not inhibited) and an SnRK2 (serine/threonine-protein kinase SAPK7-like, Solyc05g056550) was downregulated in SC, wherePP2Cs are inhibited. In addition, SnRK2s can phosphorylate b-ZIP transcription factors, which bind to the ABA-responsive element to activate ABA-responsive genes. Phosphorylated b-ZIP transcription factors are important to active ABA-responsive genes [57]. One b-ZIP transcription factor (Solyc10g076920) was significantly downregulated in the SCP *vs.* SCUP comparison, but not in the SIP *vs.* SIUP comparison. This indicated that ABA might have important regulatory roles in SI. Jasmonates are phytohormones that are essential for plant development and survival, and can induce jasmonate ZIM-domain proteins (JAZs) to be degraded through the ubiquitin/proteasome pathway, mediated by the SCF^COI1^ complex. In addition, JAZs negatively regulate MYC2, which is a key jasmonate responses transcriptional activator [58]. We identified a JAZ (jasmonate ZIM-domain protein 1, Solyc12g009220) and a transcription factor MYC2 (Solyc08g076930), both of which were both significantly upregulated in the SIP *vs.* SIUP comparison. The TGA family comprises key transcription factors of the salicylic acid (SA)-mediated signal transduction pathway [59]. After SI self-pollination, TGA family transcription factor (Solyc10g080410) was significantly upregulated.

## Conclusions

This is the first global transcriptome-wide comparative analysis of styles from SC and SI tomatoes using a high-throughput RNA-seq. The enriched GO term analysis of the identified DEGs showed that nine GO terms were significantly enriched in the SCP *vs.* SCUP comparison and 78 GO terms were significantly enriched in the SIP *vs.* SIUP comparison. The ethylene biosynthesis pathway of the cysteine and methionine metabolism pathway and the plant hormone signal transduction pathway play an important role in tomato SI. Further GO and KEGG analyses showed that SI self-pollination induced more ethylene production and catabolism of ABA, and SC self-pollination induced more auxin production and ABA biosynthesis. Moreover, the phytohormones ethylene, auxin and ABA play important roles by plant hormone signal transduction in tomato SI.

## Methods

### Plant materials

Tomato seeds of *S. chilense* (LA0130, SI) and *S. pimpinellifolium* (LA1585, SC) were obtained from the Charles Rick Tomato Genetics Resource Center (UC, Davis http://tgrc.ucdavis.edu/index.aspx). The seeds were germinated in peat pellets and seedlings with three to four leaves were grown on medium containing the perlite: peat (1:1) under a thermoperiod of 26/20°C (day/night) in a greenhouse. Plants were supplied with a commercial fertilizer every week. During flowering, 24 h UP and P styles (containing stigmas) (Additional file 5) were collected from *S. chilense* (LA0130) (SIUP/SIP) and *S. pimpinellifolium* (LA1585) (SCUP/SCP), respectively, and immediately frozen in liquid nitrogen and stored at −80°C for RNA extraction. Three biological replicates of each sample were collected and used for RNA extraction.

### RNA extraction and deep sequencing

Total RNA was extracted from each sample using an RNAprep pure Plant Kit (Tiangen, Beijing, China), according to the manufacturer’s protocol. The RNA concentration of each sample was measured using a NanoDrop 2000 (Thermo Scientific, Waltham, MA, USA). The RNA quality was assessed using an Agilent2200 (Agilent Technologies, Santa Clara, CA, USA).

The sequencing library for each RNA sample was prepared using a TruseqTM RNA sample prep Kit (Illumina, San Diego, CA, USA), following the manufacturer’s protocol. Briefly, mRNA was purified using poly-T oligo-attached magnetic beads (Invitrogen,Carlsbad, CA, USA) from 5 μg total RNA. The mRNA was fragmented, and the RNA fragments were reverse transcribed and amplified to double-stranded cDNA. Index adapters were then ligated to the cDNA according to the protocol of the TruseqTM RNA sample prep Kit (Illumina). The library was quantified using a TBS-380 mini-fluorometer (Picogreen, Cohasset, MA, USA). The clustering of the index-coded samples was performed on a cBot Cluster Generation System, using a TruSeq PE Cluster Kit v3-cBot-HS (Illumina), according to the manufacturer’s instructions. After cluster generation, the library preparations were sequenced on an Illumina Hiseq 2500 platform and a sequence length of 2*101 bp paired-end reads were generated.

### Filtering raw reads and mapping

The raw reads were pass-filtered using the Trimmomatic tool [60] and then used for mapping. The reference tomato genome and gene model annotation files were downloaded from the genome website (http://solgenomics.net/) directly. The paired-end clean reads were aligned to the reference tomato genome using Tophat [61] and the mapped reads were counted with using HT-seq [62].

### Identification of DEGs

Gene expression levels were estimated as RPKM [63]. Differential expression analysis of SCUP/SCP groups and SIUP/SIP groups was performed using the DESeq R package (1.10.1), which provides statistical routines for determining differential expression in digital gene expression data using a model based on the negative binomial distribution. After statistical analysis, the DEGs were identified using significance analysis by t-tests, with a P-value < 0.05 and at least two-fold changes (either up- or downregulation) being considered significant.

### GO analysis

The blast2go [64] program was used to obtain GO annotations for all identified genes. GO functional classification was performed using the WEGO online tool [65] to gain an understanding of the distribution of gene functions at the macro level. GO is the key functional classification of NCBI, which was applied to analyze the functions of the DEGs [66,67]. GO enrichment analysis of DEGs was implemented using SEA [68], in which Fisher’s exact test and a χ^2^ test were used to classify the GO categories; the FDR was calculated to correct the P-value [69,70]. P-values for the GOs of all the DEGs were computed. The significant GO terms were defined as having a P-value < 0.05 and an FDR < 0.05.

### Pathway analysis

KEGG is a database resource for understanding high-level functions and utilities of biological systems, such as cells, organisms and ecosystems, from molecular-level information, especially large-scale molecular datasets generated by genome sequencing and other high-through put experimental technologies (http://www.genome.jp/kegg/). KEGG pathway analysis was used to identify the significant pathways involving the DCEGs [71-73]. Fisher’s exact test and a χ^2^ test were used to identify significant pathways (P-value < 0.05 and FDR < 0.05) [74-76]. We used the KEGG Orthology Based Annotation System (KOBAS) software to test the statistical enrichment of DEGs in KEGG pathways.

### Availability of supporting data

The data sets supporting the results of this article are available in the Gene Expression Omnibus repository under accession no GSE67654 (http://www.ncbi.nlm.nih.gov/geo/query/acc.cgi?acc=GSE67654) [77].

## Additional files

Additional file 1: Table S1. RNA-seq of data of all counts for SI and SC compared with self-pollinated and unpollinated styles. Additional file 2: Table S2. List of differentially expressed genes for SI and SC compared with self-pollinated and unpollinated styles. Additional file 3: Table S3. GO analysis of differentially expressed genes for SI and SC compared with self-pollinated and unpollinated styles. Additional file 4: Table S4. Pathway analysis differentially expressed genes for SI and SC compared with self-pollinated and unpollinated styles. Additional file 5: Figure S1. The structure of the tomato pistil. Red lines show the cutting position of a style containing a stigma.

## Abbreviations

SC: Self-compatibility
SI: Self-incompatibility
ncRNA: Non-coding RNAs
SAGE: Serial Analysis of Gene Expression
EST: Expressed sequence tag
MPSS: Massively parallel signature sequencing
RNA-seq: mRNA sequencing
GO: Gene ontology
GB: Gigabases
SCUP: SC unpollination
SCP: SC self-pollination
SIUP: SI unpollination
SIP: SI self-pollination
UP: Unpollination
P: Pollination
RPKM: Reads per kilobase of exon region in a given gene per million mapped reads
DEGs: Differentially expressed genes
FC: Fold-change
SEA: Singular enrichment analysis
FDR: False discovery rate
KEGG: Kyoto encyclopedia of genes and genomes
AdoMet: S-adenosyl-L-methionine
ACC: 1-aminocyclopropane-1-carboxylate
ABA: Abscisic acid
ACO: 1-aminocyclopropane-1-carboxylate oxidase
ACS: 1-aminocyclopropane-1-carboxylate synthase
SAM: S-adenosyl methionine synthase
ETR: Ethylene receptor
EIN3/EIL: Ethylene-insensitive protein 3
ERF: Ethylene-responsive transcription factor
EBF1/EBF2: Ein3-binding f-box protein1/2
IAA: Indole-3-acetic acid
AUX1 LAX: Auxin influx carrier
Aux/IAA: Auxin/indole-3-acetic acid
SAUR: Small auxin-up RNA
JA: Jasmonic acid
RBX1: RING-box protein 1
Cul1: Cullin 1
Skp1: S-phase kinase-associated protein 1
F-box: F-box protein
PYR/PYL: Pyrabactin resistance/pyrabactin resistance-like
PP2Cs: Type 2C protein phosphatases
SnRK2: SNF1-related protein kinase subfamily 2
JAZs: Jasmonate ZIM-domain protein
KOBAS: KEGG Orthology Based Annotation System
