# Supplementary figures and images for: Dissection of the style’s response to pollination using transcriptome profiling in self-compatible (Solanum pimpinellifolium) and self-incompatible (Solanum chilense) tomato species
Source: BMC Plant Biol. 2015 May 15;15:119. doi: 10.1186/s12870-015-0492-7 (PMC4431037; doi:10.1186/s12870-015-0492-7)

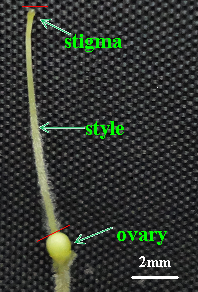

Supplement: Additional file 5: Figure S1. — The structure of the tomato pistil. Red lines show the cutting position of a style containing a stigma. [file 12870_2015_492_MOESM5_ESM.tif]
